# Supplementary material for: Cognitive task information is transferred between brain regions via resting-state network topology
Source: Nat Commun. 2017 Oct 18;8:1027. doi: 10.1038/s41467-017-01000-w (PMC5715061; doi:10.1038/s41467-017-01000-w)
Supplement: Supplementary file 1 — Supplementary Information [file 41467_2017_1000_MOESM1_ESM.pdf]

Supplementary Figures

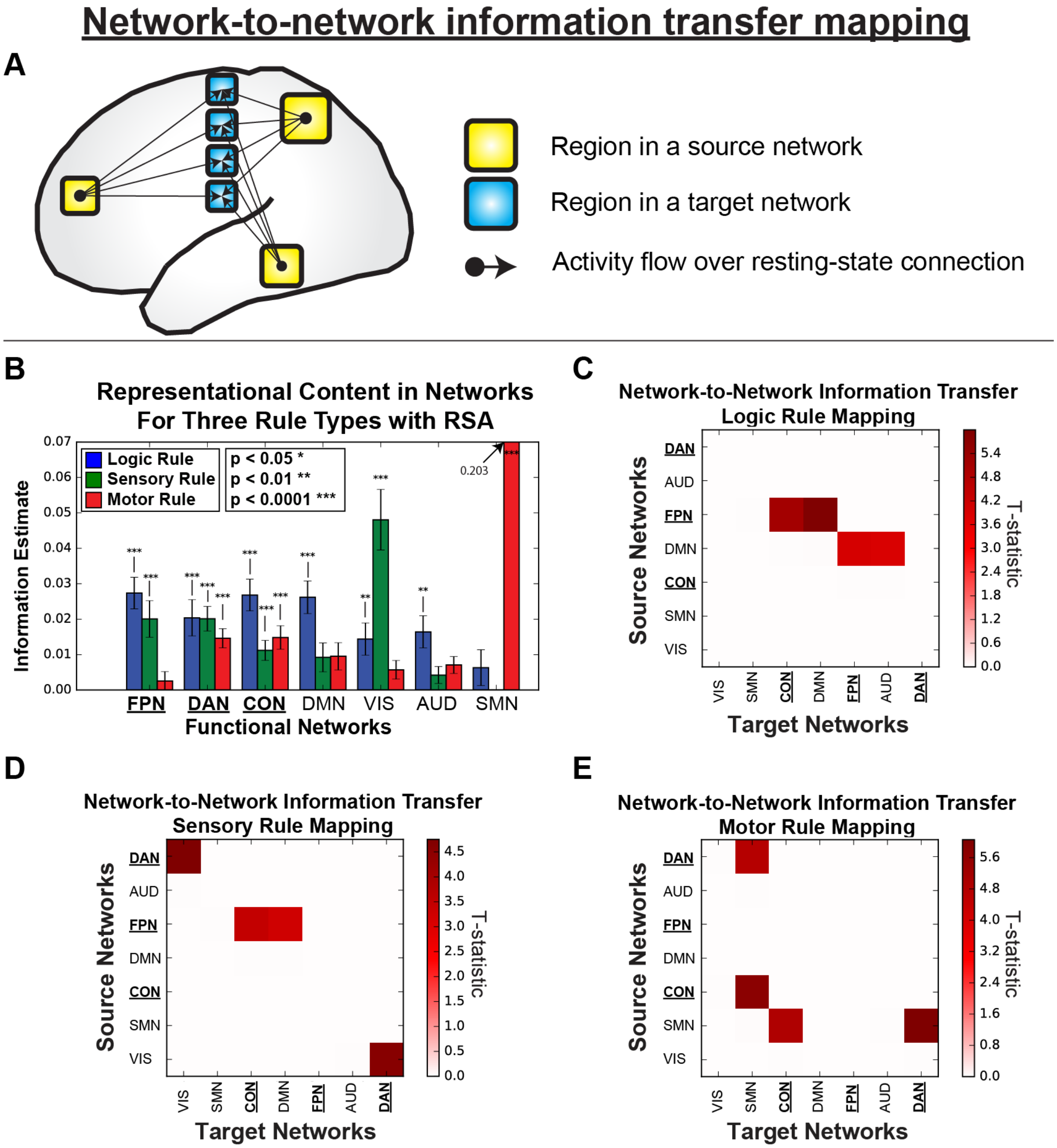

Supplementary Figure 1. Task information persist in functional networks and are transferred between networks via network-to-network information transfer mapping. All reported results were statistically significant p<0.05 (FWE-corrected). **A)** Network-to-network information transfer mapping uses the network-level activation pattern (using the mean activations of brain regions) and the region-to-region resting-state FC topology to predict the network-level activation pattern of another functional network. **B)** Information estimates of task-rule information across three rule domains prior to performing information transfer mapping. The seven networks contain statistically significant decodable representations of at least one rule domain using a cross-validated representational similarity analysis approach. In particular, the

SMN contains the highest information estimate for motor task rules. In addition, most networks contain logic rule information, suggesting that abstract rule representations were highly distributed across cortical networks. **C)** Network-to-network information transfer mapping of logic rules. As in Fig. 6, functional networks along the rows indicate the activation patterns that were projected to the networks indicated along the columns. Colors indicate the T-statistic from a one-sided t-test against 0. The transfer of logic rule information was distributed among other domain-general networks, such as the CON and DMN. **D)** Network-to-network information transfer mapping of the sensory rules. Sensory rule information is transferred between the FPN and other domain-general networks (DMN, CON), as well as from VIS and the DAN. **E)** Network-to-network information transfer of motor rules. Information transfer mapping of motor rule representations occurs between the DAN <-> SMN, CON <-> SMN.

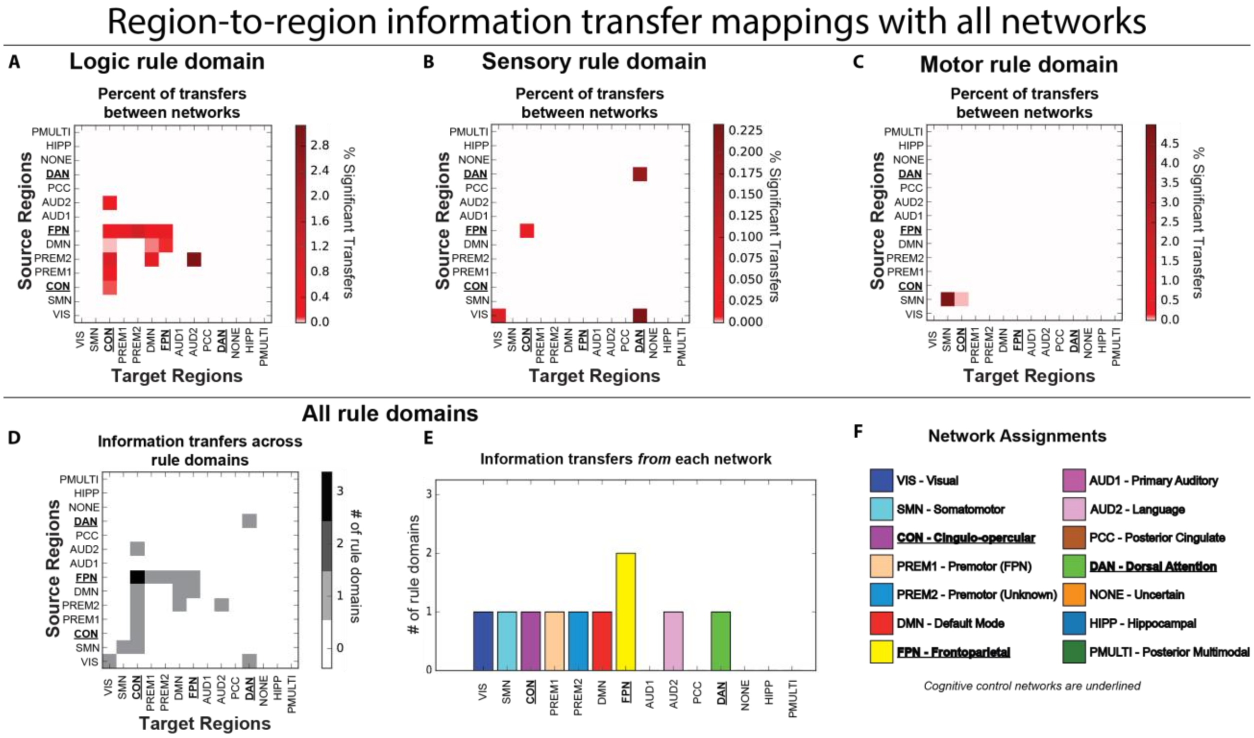

Supplementary Figure 2. Information transfer mappings between all pairs of regions for all defined functional networks. All reported results were statistically significant at p<0.05 (FWE-corrected). Here, we show a superset of the summary results shown in Fig. 6B,D,F, including significant information transfer results for all 14 functional networks. While results for all regions (belonging to all 14 functional networks) are shown in panels Fig. 6A,C,E, functional networks that were not well-defined by previous network partitions were not included in Fig. 6B,D,F. Note that the 7 functional networks not included in Fig. 6 are the seven smallest networks, each consisting of fewer than 20 parcels. **A)** Percent of significant region-to-region information transfers for all 14 network definitions for the logic rule domain. **B)** Percent of significant region-to-region information transfers for all 14 network affiliations for the sensory rule domain. **C)** Percent of significant region-to-region information transfers for all 14 network affiliations for the motor rule domain. **D)** Significant information transfers between regions for all 14 network affiliation across rule domains, derived in the same way as data in Fig. 6G. Despite including all functional networks, we found that transfers between the FPN and the CON were still the only transfers between a pair of networks that consistently transferred information across two rule domains. **E)** We assessed whether a network was consistently involved in sending task rule information (as a source region) across each rule domain. We found that regions in the FPN consistently transferred information across two rule domains. **F)** Network assignments and color definitions for all 14 functional networks. Here, we attribute functional names for all 14 networks. Color schemes are consistent with colorings shown on the anatomical surface in Fig. 3A.

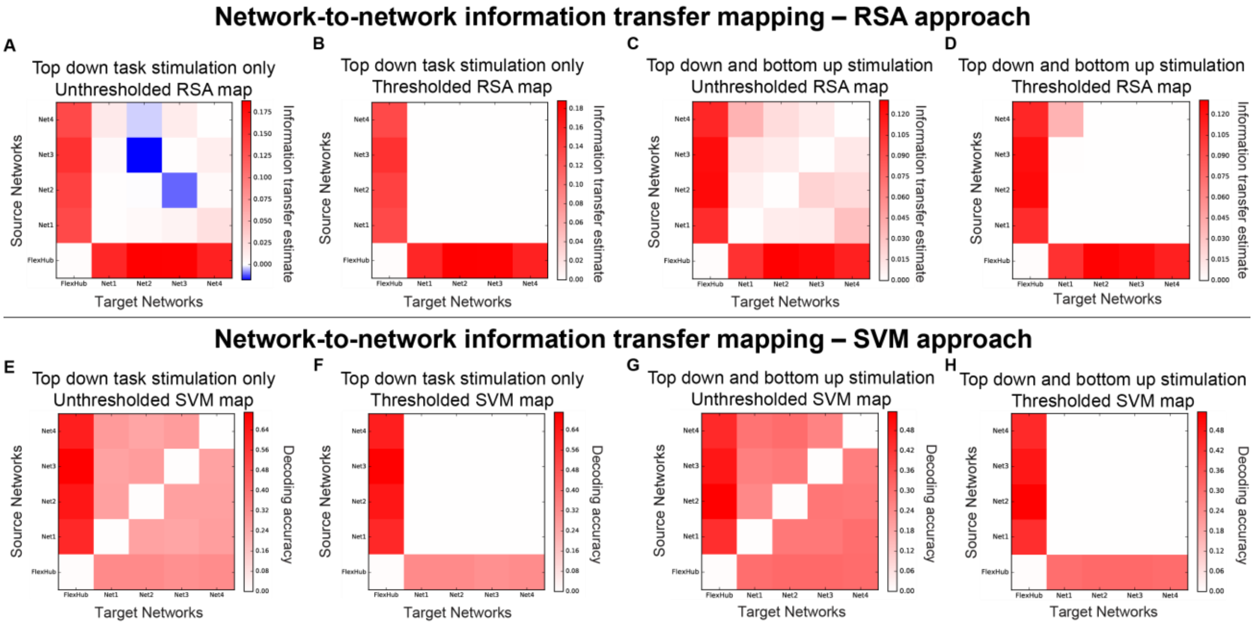

Supplementary Figure 3. Computational validation of information transfer mapping with different task stimulation patterns and decoding approaches. We simulated an additional 35 subjects to test whether information transfer mapping would be consistent across different task stimulation patterns and decoding approaches. **A)** We performed the same analysis as depicted in Fig. 4, where we simulated cognitive control task rules by stimulating regions in the hub network for four distinct task-rule conditions. We depict the uncorrected information transfer estimates for every network-to-network configuration using the information transfer mapping procedure described in Fig. 1C. **B)** Thresholded map of panel Supplementary Figure 3A. For every network-to-network information transfer mapping, we performed an across subject, one-sided t-test against 0. Statistical significance is assessed using FWE-corrected p-values of $p<0.05$. **C)** We simulated a task that combined both top down stimulation (e.g., mimicking task-rule encoding) and bottom up stimulation in local networks (e.g., mimicking stimuli presentations). This task also included four distinct task conditions, where each condition stimulated a subset of regions in the hub network along with a subset of regions in a local network simultaneously. Each task condition stimulated a subset of regions in a different local network. **D)** Thresholded map of panel C, using FWE-corrected p-values of $p<0.05$. While the pattern of information transfer was largely the same, information transfer of both top down and bottom up stimulation was more disperse than top down stimulation only. **E-H)** We performed the group analyses on the same exact data (see Supplementary materials) as Supplementary Figures 3A-D but instead of using an RSA approach (i.e., predicted-to-similarity analysis; Fig. 1C), we used SVMs (training on predicted activation patterns and testing on held-out actual activation patterns). Note that Supplementary Fig. 3F has qualitatively identical results as in our computational validation results (Fig. 4E) using representational similarity analysis. For panels F and H, statistical significance was assessed using one-sided t-tests against chance (25% chance) for a four-way task condition classification. Thresholds were applied using an FWE-corrected $p<0.05$. All panels show the raw effect sizes.

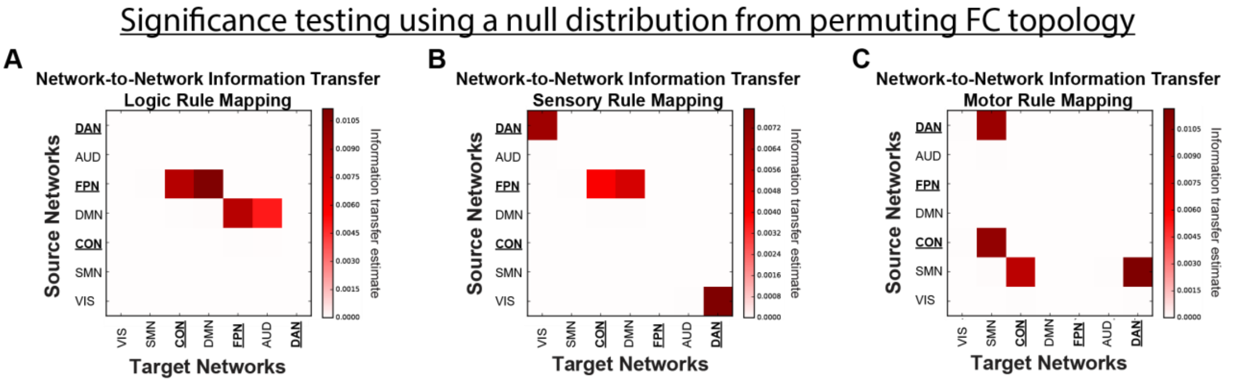

Supplementary Figure 4. Network-to-network information transfer mapping depends on precise FC topology between pairs of networks. All reported results were statistically significant at $p<0.05$ (FWE-corrected). To ensure that information transfer mapping between networks depended on the precise FC topology between pairs of networks, we generated a null distribution of information transfers by permuting the inter-region FC patterns between pairs of networks prior to performing the network-to-network information transfer procedure. For each network-to-network information transfer mapping, 1000 FC permutations were conducted. Significant results demonstrate that the information transfer depended on the precise network-to-network FC topology. This analysis demonstrates that the results obtained using parametric statistical testing (Supplementary Fig. 1) depend on the precise inter-region FC patterns between pairs of networks, as results from the parametric and non-parametric tests are virtually identical. Color maps represent the group averaged information transfer estimate, since no t-statistic is available in the null distribution.

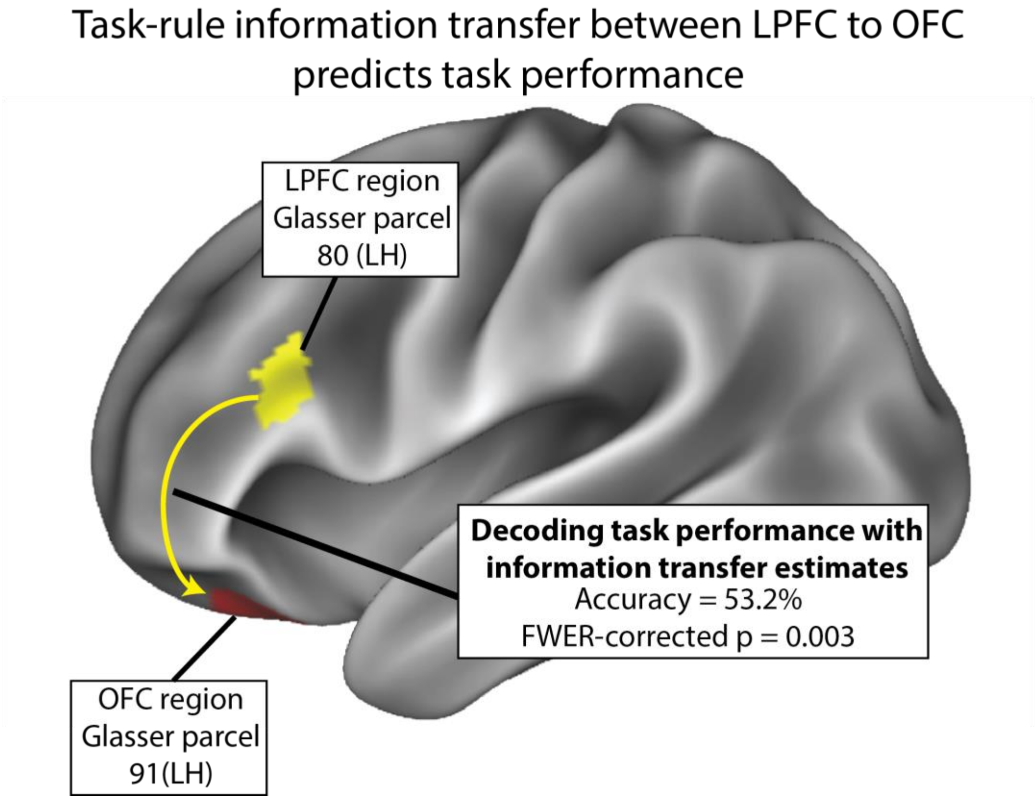

Supplementary Figure 5. The behavioral relevance of cognitive task information transfer. We found that task-rule information transfer between two FPN regions could decode miniblock task performance significantly above chance. We constructed a decoding model using multiple logistic regression to decode task performance in a held-out miniblock by fitting to the logic, sensory, and motor information transfer estimates across miniblocks. When transformed into the OFC region's spatial dimensions, task-rule information in the LPFC region could predict a miniblock's task performance significantly above chance, suggesting that the transfer of task-rule information between these regions is relevant for task performance.

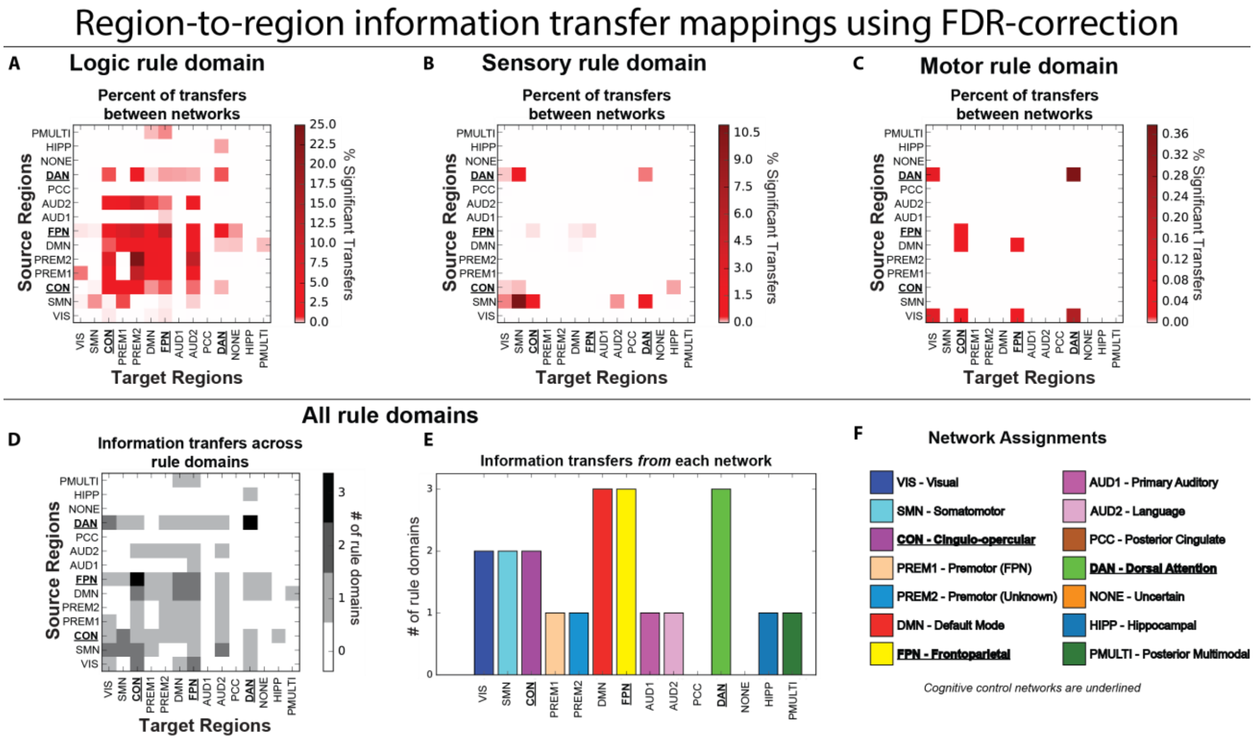

Supplementary Figure 6. Information transfer mappings between all pairs of regions using an FDR-corrected threshold for all defined functional networks. Due to the conservative nature of FWE correction for multiple comparisons correction, we also report the same results from Fig. 6 and Supplementary Figure 2 using an FDR-corrected p-value of p<0.05. Using FDR-correction, we found that statistically significant task-rule information transfers were much more distributed than with FWE-correction, particularly with logic rule transfers. **A)** Percent of significant region-to-region information transfers for all 14 network definitions for the logic rule domain. **B)** Percent of significant region-to-region information transfers for all 14 network affiliations for the sensory rule domain. **C)** Percent of significant region-to-region information transfers for all 14 network affiliations for the motor rule domain. **D)** Significant information transfers between regions for all 14 network affiliation across rule domains, derived in the same way as data in Fig. 6G. **E)** We assessed whether a network was consistently involved in sending task rule information (as a source region) across the three rule domains. We find that with an FDR-corrected threshold of p<0.05, the FPN, DAN, and DMN all contain regions that transfer information across all three rule domains. **F)** Network assignments and color definitions for all 14 functional networks. Here, we attribute functional names for all 14 networks. Color schemes are consistent with colorings shown on the anatomical surface in Fig. 3A.

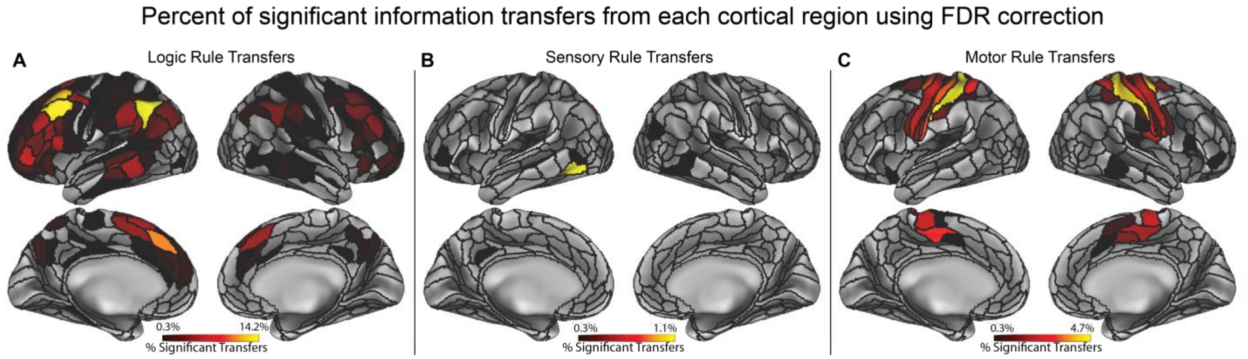

Supplementary Figure 7. Percent of significant information transfers from each cortical region using an FDR-corrected threshold. Due to the conservative nature of FWE correction for multiple comparisons correction, we also report results from Fig. 7 using an FDR-corrected threshold of $p<0.05$. **A)** Percent of statistically significant information transfers from each region for the logic rule domain. Percentages were computed by taking the number of significant transfers from each region, and dividing it by the total number of possible transfers from that region (359 other regions). **B)** Percent of statistically significant information transfers from each region for the sensory rule domain. **C)** Percent of statistically significant information transfers from each region for the motor rule domain.

Supplementary Methods

We provide Supplementary Methods for several of our Methods subsections below. For completeness, we have included redundant text for the Methods subsections that contain additional information. However, subsections for which there is no additional information (e.g., the "Participants" subsection) are not included in the Supplementary Methods.

Behavioral paradigm

We used the Concrete Permuted Rule Operations (C-PRO) paradigm (Fig. 2), which is a modified version of the original PRO paradigm introduced in Cole et al., (2010)[1]. Briefly, the C-PRO cognitive paradigm permutes specific task rules from three different rule domains (logical decision, sensory semantic, and motor response) to generate dozens of novel and unique task sets. This creates a condition-rich dataset in the task configuration domain akin in some ways to movies and other condition-rich datasets used to investigate visual and auditory domains[2–4]. The primary modification of the C-PRO paradigm from the PRO paradigm was to use concrete, sensory (simultaneously presented visual and auditory) stimuli, as opposed to the abstract, linguistic stimuli in the original paradigm. Visual stimuli included either horizontal or vertical oriented bars with either blue or red coloring. Simultaneously presented auditory stimuli included continuous (constant) or non-continuous (non-constant, i.e., "beeping") tones beeps presented at high (3000Hz) or low (300Hz) frequencies. Fig. 2 demonstrates two example task-rule sets for "Task 1" and "Task 64". The paradigm was presented using E-Prime software version 2.0.10.353[5].

Each rule domain (logic, sensory, and motor) consisted of four specific rules, while each task set was a combination of one rule from each rule domain (Fig. 2). The sensory rules specified the audiovisual features to attend to (e.g., "is it vertical?" for visual decisions, or "is it high-pitch?" for auditory decisions). The logic rules specified how to respond based on the pair of stimuli presentations (e.g., "if both are vertical" or "if either are vertical"). Finally, the motor rules specified which button to press, which depended on the answer to the logic rule. For "true" outcomes, subjects were asked to respond with the motor rule presented in the task-rule set; for "false" outcomes, subjects were asked to respond with the other finger on the same hand.

A total of 64 unique task sets (4 logic rules x 4 sensory rules x 4 motor rules) were possible, and each unique task set was presented twice for a total of 128 task miniblocks. Identical task sets were not presented in consecutive blocks. Each task miniblock included three trials, each consisting of two sequentially presented instances of simultaneous audiovisual stimuli. A task block began with a 3925ms instruction screen (5 TRs), followed by a jittered delay ranging from 1570ms to 6280ms (2 – 8 TRs; randomly selected). Following the jittered delay, three trials were presented for 2355ms (3 TRs), each with an inter-trial interval of 1570ms (2 TRs). A second jittered delay followed the third trial, lasting 7850ms to 12560ms (10-16 TRs; randomly selected). A task block lasted a total of 28260ms (36 TRs). Subjects were trained on four of the 64 task-rule sets for 30 minutes prior to the fMRI session. The four practiced rule sets were selected such that all 12 rules were equally practiced. There were 16 such groups of four task sets possible, and the task sets chosen to be practiced were counterbalanced

across subjects. Subjects' mean performance across all trials performed in the scanner was 85% (median=86%) with a standard deviation of 8% (min=66%; max=96%). All subjects performed statistically above chance (25%).

Network assignment of Glasser et al. (2016) parcels

Partitioning of the parcels (regions) into networks was based on the procedure used in Cole et al. (2014; see Supplementary Information). Specifically, we used the Louvain locally-greedy algorithm[9,10] for community detection. Data from the publically available Washington University-Minnesota Human Connectome Project "HCP100" dataset were used (N=100). Similar preprocessing procedures as used for the primary dataset were applied to the HCP100 dataset. Specifically, in addition to minimal preprocessing[7], we ran a GLM nuisance regression using white matter, ventricles, and motion regressors (and their first derivatives). Global signal regression, motion scrubbing, and temporal filtering were not used. For each subject, all four resting state runs were concatenated and FC was estimated using standard Pearson correlations. The FC matrices were averaged across subjects to generate a group-mean resting-state FC matrix.

We searched over two free parameters to find a community partition for the group-mean resting-state FC matrix. The first parameter was the density threshold, whereby weak connections (based on the absolute value of FC strengths) were removed prior to running the community detection algorithm. The second parameter was the structural resolution parameter, which can be used to tune the number of communities identified in the FC matrix. The parameter search was conducted across combinations of these two parameters (density of 40% to 100% in increments of 5%, and resolution of 0.8 to 3 in increments of 0.05), with two criteria: 1) there should be a peak of partition similarity (z-score of the Rand coefficient)[11] among adjacent locations in this two-dimensional parameter space, and 2) there should be distinct communities corresponding to visual, auditory, dorsal attention, default-mode, and motor/tactile systems (given decades of neuroscience research demonstrating their existence). Approximate locations of these systems were based on standard neuroscientific knowledge of these systems (given their strong establishment in the literature), in addition to their identification using resting-state FC in previous reports[12–14]. A five-community partition had the highest nearest-neighbor similarity in parameter space, but this did not separate out the auditory system. The next-highest nearest-neighbor similarity peak (density = 100%, resolution = 1.2) with distinct communities corresponding to auditory, visual, dorsal attention, default-mode, and motor/tactile systems was a 14-community partition. This partition was then visualized using Connectome Workbench software (Fig. 3A). Labels were assigned to the seven most replicated networks identified using resting-state FC[12–14]. Colors were assigned to networks based on the colors used by Power et al. (2011).

Neural network model

To validate our information transfer estimation approach we constructed a simple dynamical neural network model with similar network topological properties identified in our empirical fMRI data. We constructed a neural network with 250 regions, each of which were clustered into one of five network communities (50 regions per community).

Regions within the same community had a 35% probability of connecting to another region (i.e., 35% connectivity density), and regions not assigned to the same community were assigned a connectivity probability of 5% (i.e., 5% out-of-network connectivity density). We selected one community to act as a "network hub", and increased the out-of-network connectivity density of those regions to 20% density. We then applied Gaussian weights on top of the underlying structural connectivity to simulate mean-field synaptic excitation between regions. These mean-field synaptic weights were set with a mean of $1.0/\sqrt{K}$ with a standard deviation of $0.2/\sqrt{K}$, where K is the number of synaptic inputs into a region such that synaptic input scales proportionally with the number of inputs. This approach was recently shown to be a plausible rule in real-world neural systems based on *in vitro* estimation of between-neuron synaptic-weight-setting rules[16].

To simulate network-level firing rate dynamics, as similar to Stern et al. (2014), region x_i's dynamics for $i = 1 \dots 250$ obeyed the equation

$$\frac{dx_i}{dt}\tau_i = -x_i(t) + s\,\phi\left(x_i(t)\right) + g\left(\sum_{j \neq i}^{N} W_{ij}\,\phi\left(x_j(t)\right)\right) + I_i(t) \qquad (3)$$

We define the transfer function ϕ as the hyperbolic tangent, x_j the dynamics of region $j = 1 \dots 250$ for $i \neq j$, $I_i(t)$ the input function (e.g., external spontaneous activity alone or both spontaneous activity and task stimulation) for $i = 1 \dots 250$, W the underlying synaptic weight matrix, s the local coupling (i.e., recurrent) parameter, g the global coupling parameter, and τ_i the region's time constant. For simplicity, we set $s = g = 1$ and $\tau_i = 10$ ms, though we show in a previous study[8] that the activity flow mapping breaks down for parameter regimes $s \gg g$.

We first simulated spontaneous activity in our model by injecting Gaussian noise (parameter $I_i(t)$; mean of 0.0, standard deviation 1.0). Numerical simulations were computed using a Runge-Kutta second order method with a time step of dt=10 ms. We ran our simulation for 600 seconds (10 minutes). To simulate resting-state fMRI, we then convolved our time series with the SPM canonical hemodynamic response function and down sampled to a 1 second TR, resulting in 600 time points. We then computed resting-state FC using multiple linear regression. To replicate the empirical data, we computed the BGC of the resting-state data (as in the empirical data) to validate that widespread out-of-network connectivity was preserved from synaptic to FC.

To model task-evoked activity, we simulated four distinct task conditions by injecting stimulation into four randomly selected but distinct sets of twelve regions in the hub network. Stimulation to the hub network was chosen to mimic four distinct top-down, cognitive control task rules. Task stimulation coincided with spontaneous activity (e.g., for time points t during a task, I(t) = spontaneous activity at t + 0.5 constant task stimulation). We ran each task for 20 blocks, where each block lasted for 100 seconds. Each block contained five trials, each lasting for five seconds with an inter-trial interval of 15 seconds. In total, each task condition contained 100 task trials, with 500 seconds per task total. We then convolved these task time series with the SPM canonical hemodynamic response function and down sampled to 1-second TRs, as in the resting-state simulation. We simulated 30 subjects worth of data, and generated figures using group t-tests and controlled for multiple comparisons using FWE-correction permutation tests[15].

We validated the usefulness of the model for characterizing hub-related dynamics by testing whether estimated resting-state FC preserved the hub network's higher out-of-network intrinsic FC (specified by its underlying synaptic connectivity) by computing each network's BGC. BGC was computed in the same way as in the empirical data (see equation 2) for each of the network model's communities. For each of the five networks, we compared the BGC between each network using a cross-subject t-test. We corrected for multiple comparisons using FWE permutation tests[15] and significance was assessed with an FWE-corrected $p < 0.05$ threshold.

To perform network-to-network information transfer mapping in the model, we used the task-evoked activity (estimated by standard GLM beta estimates), and performed the information transfer mapping procedure between networks of regions using the resting-state FC matrix obtained via multiple linear regression. Network-to-network information transfer mapping is computationally identical to region-to-region information transfer mapping, and is described below. The information transfer mapping matrix (Fig. 4E) was obtained using an FWE-corrected threshold of $p < 0.05$.

We primarily focused on stimulating the hub network to mimic top-down processes, since our empirical results focused on task-rule manipulations irrespective of stimuli presentations and motor responses. However, to demonstrate the generality with which information transfer can occur, we performed an additional set of simulations that focused on demonstrating that information transfer occurs with simultaneous top-down (hub network) and bottom-up (local network) stimulation. Using the same parameters as in the original simulation, we first replicated the same results as in Fig. 4E with hub network stimulation only (i.e., top-down control). To simulate top-down and bottom-up activation we simulated four task conditions by injecting activity into four sets of regions. For each task condition, we simultaneously injected two sets of 12 regions; one set of 12 regions in the hub network (mimicking top-down activity), and one set of 12 regions in a local network (mimicking bottom-up activity). Each task condition stimulated a set of regions belonging to a different local network and a distinct set of regions in the hub network. Aside from task stimulation, all other model and simulation parameters were kept the same from the simulation result in Fig. 4.

Our results were highly similar to the previous results, demonstrating that in both the top-down-only task and the simultaneous top-down and bottom-up task, information transfers between the hub and local networks were the strongest (Supplementary Fig. 3A,C). However, statistical testing demonstrated that some local-network-to-local-network information transfers were significant (after correcting for multiple comparisons; Supplementary Fig. 3D,H). We believe these effects are likely due to the existence of random (albeit sparse) connections between local networks. We also show that the predicted-to-actual similarity analysis portion of the information transfer procedure (described below) can be substituted with support vector machine (SVM) classification (Supplementary Fig. 3E-H; see below for details).

Computing baseline information estimates for regions and networks

To compute the baseline (i.e., unrelated to FC) information content at the region level (Fig. 5), we performed a within-subject, cross-validated multivariate pattern analysis using representational similarity analysis for every Glasser et al. (2016) parcel (using the vertex-level multivariate activation pattern within each parcel). We estimated

task-activation beta coefficients separately for each vertex within a region, and separately for each miniblock. Note that each miniblock was associated with a specific task-rule condition for each rule domain. Mathematically, we defined IE_B , the information estimate of region B, as

$$\boldsymbol{IE_B} = \boldsymbol{Match_B} - \boldsymbol{Mismatch_B} \qquad (4)$$

where **Match**$_B$ and **Mismatch**$_B$ correspond to the averaged Spearman rank correlation for matched and mismatched conditions, respectively. Specifically, we define **Match**$_B$ and **Mismatch**$_B$ as

$$\boldsymbol{Match_B} = \frac{\sum_{k=1}^{K} scorr(\boldsymbol{B}_k, \boldsymbol{B}_{match})}{K} \qquad (5)$$

$$\boldsymbol{Mismatch_B} = \frac{\sum_{k=1}^{K}\left[\sum_{n=1}^{N}\left(scorr(\boldsymbol{B}_k, \boldsymbol{B}_{mismatch_n}\right)/N\right]}{K} \qquad (6)$$

where K corresponds to the total number of miniblocks (in this paradigm, 128 miniblocks), $scorr$ corresponds to a Fisher z-transformed Spearman's rank correlation between two activation vectors, $\boldsymbol{B}_k$ is the activation pattern in region B during block k, $\boldsymbol{B}_{match}$ is the task-rule condition prototype (obtained by averaging across blocks of the same condition, holding out block k) of region B's activation pattern for which block k's condition matches, and $\boldsymbol{B}_{mismatch_n}$ as the task-rule condition prototypes for which block k's condition does not match. (In the present study $N = 3$, since each rule dimension has four task-rule conditions, and for a given miniblock there's one match and three mismatched conditions.) To avoid circularity, we performed a leave-four-out cross-validation scheme, holding out a miniblock of each task-rule. This ensured that miniblock $\boldsymbol{B}_k$ was not included in constructing the condition prototype $\boldsymbol{B}_{match}$ and that condition prototypes were each constructed using the same number of miniblocks. Prior to running the representational similarity analysis, all blocks were spatially demeaned to increase the likelihood that the representations we were identifying was a multivariate regional pattern (rather than a change in region-level mean activity). Use of Spearman's rank correlation also reduced the likelihood that the identified multivariate representation patterns were driven by mean activity changes or a small number of outlier values.

Statistical significance was assessed by taking a one-sided group t-test against 0 for each region's information estimate across subjects, since a greater than 0 difference of matches versus mismatches indicated significant representation of specific task rules. All p-values were corrected for multiple comparisons across the 360 parcels using FWE-correction with permutation tests[15], and significance was assessed using an FWE-corrected threshold of $p<0.05$.

For network-level information estimates (Supplementary Fig. 1B), the same cross-validated representational similarity analysis procedure was conducted for the seven functional networks separately across the three rule domains, using region-level representations within each of the networks. Region-level beta estimates were obtained for every block by fitting the same GLM model as described above to every region separately. All p-values were FWE-corrected for multiple comparisons across seven networks with permutation tests[15], and significance was assessed using an FWE-corrected $p<0.05$.

Region-to-region information transfer mapping

We extended the original activity flow mapping procedure as defined in Cole et al. (2016)[8] (Fig. 1A) to investigate transfer of task-related information between pairs of brain regions using vertex-wise activation patterns (i.e., region-to-region activity flow mapping; Fig. 1B). The original activity flow mapping approach predicted the activity level of a single held-out region using the weighted sum of the task-evoked activity of all other regions. These activation estimates were obtained using a standard fMRI general linear model (GLM). The weights in the weighted sum were based on the resting-state FC from the source regions to the held-out region. The region-to-region activity flow mapping procedure developed here is computationally similar. However, instead of predicting the activity of a single held-out region based on all other regions, we predicted the activity of the vertices of a held-out target region based on the vertices within a source region. Mathematically, we define region-to-region activity flow mapping between regions A and B as

$$\overline{\boldsymbol{B}}_k = \boldsymbol{A}_k \bullet W_{RSFC} \qquad (7)$$

where $\overline{\boldsymbol{B}}_k$ corresponds to the predicted activation pattern vector for the target region B, $\boldsymbol{A}_k$ corresponds to region A's activation pattern vector (i.e., the source region), W_{RSFC} corresponds to the vertex-to-vertex resting-state FC between regions A and B, and the operator • refers to the dot product. This formulation allowed us to map activation patterns in one region's spatial dimension to the spatial dimension of another region.

To test the extent that task representations are preserved in the region-to-region multivariate predictions, we quantified how much information transfer occurred between the two regions. Briefly, information transfer mapping comprises three steps, illustrated in Fig. 1C: (1) Region-to-region (or network-to-network) activity flow mapping; (2) A cross-validated representational similarity analysis between predicted activation patterns and actual, held-out activation patterns; (3) Information classification/decoding by computing the difference between matched condition similarities and mismatched condition similarities. This final step produces an information transfer estimate.

Mathematically, our information transfer estimate was derived using almost the exact formulation as our information estimate formula. Specifically, we defined information transfer between regions A and B, or $\boldsymbol{ITE}_{AB}$, as

$$\boldsymbol{ITE}_{AB} = \boldsymbol{Match}_{AB} - \boldsymbol{Mismatch}_{AB} \qquad (8)$$

where **Match**$_{AB}$ and **Mismatch**$_{AB}$ corresponds to the averaged Spearman rank correlation for matched and mismatched conditions using the source region A, respectively. Similarly to equations 6 and 7, we define **Match**$_{AB}$ and **Mismatch**$_{AB}$ as

$$\boldsymbol{Match}_{AB} = \frac{\sum_{k=1}^{K} scorr(\overline{\boldsymbol{B}}_k, \boldsymbol{B}_{match})}{K} \qquad (9)$$

$$\boldsymbol{Mismatch}_{AB} = \frac{\sum_{k=1}^{K}\left[\sum_{n=1}^{N}\left(scorr(\overline{\boldsymbol{B}}_k, \boldsymbol{B}_{mismatch_n}\right)/N\right]}{K} \qquad (10)$$

where K corresponds to the total number of miniblocks, $scorr$ corresponds to a Fisher z-transformed Spearman's rank correlation between two vectors, $\overline{\boldsymbol{B}}_k$ as the predicted activation pattern in the target region B (using region A's activation pattern) for block k, $\boldsymbol{B}_{match}$ as the condition prototype (obtained by averaging across blocks of the same condition, holding out block k) of the target region B's actual activation pattern for which block k's condition matches, and $\boldsymbol{B}_{mismatch_n}$ as the condition prototypes for which block k's condition does not match. (In the present study $N = 3$, since each rule dimension has four task-rule conditions.) As with the previously defined information estimate, we

performed a leave-four-out cross-validation scheme, holding out a miniblock of each task-rule. This ensured that the actual activation pattern $\boldsymbol{B}_k$ of the predicted miniblock $\overline{\boldsymbol{B}}_k$ was not included in constructing the condition prototype $\boldsymbol{B}_{match}$. Prior to running the representational similarity analysis, all blocks were spatially demeaned to increase the likelihood that the representation we were identifying was a multivariate regional pattern (rather than a change in region-level mean activity). This formulation allowed us to quantify how much "information transfer" occurred between two regions by comparing the predicted activation pattern in the target region to the actual activation pattern in the target region across all cross-validation folds.

We also demonstrate that the predicted-to-actual similarity analysis in our information transfer mapping procedure can be substituted with an SVM decoding scheme. Specifically, we show in our computational model that we could train a linear classifier on the target region's predicted activation patterns that could decode the actual, activation patterns in that target region (Supplementary Figures 3E,F). We used the same leave-four-out cross-validation scheme as above to obtain these results, and we find that the information transfer mapping results with SVM decodings (Supplementary Figure 3F) are identical to using representational similarity analysis (Fig. 4E).

Note that information decoding was performed on the cortical surface, using vertices rather than voxels. This vertex-wise approach has been shown to provide better multivariate classifications than voxel-wise information decoding[17], likely because surface analyses better reflect the underlying cortical anatomy.

Information transfer mapping was performed within subject between every pair of regions in the Glasser et al. (2016) atlas (360 regions in total). The results of this approach between all region pairs were then visualized via a 360-by-360 matrix (a total of 129,240 region-to-region mappings), where the regions along rows (source regions) indicated the activation patterns used to map onto a target region's activation pattern, which was indicated along the columns (Fig. 6B,D,F). Statistical tests were performed using a group one-sided t-test ($t > 0$) for every pair-wise mapping. A one-sided t-test was appropriate here given that our hypotheses were implicitly one-sided, since any significant deviation above 0 indicated a significantly higher matched versus mismatched correlation between predicted-to-actual activation patterns (i.e., the information transfer estimate). Our use of mismatched correlations as a baseline ensured that any positive information transfer estimates was a result of a task-rule-specific representation, rather than a task-general effect. Any information estimate that was not significantly greater than 0 indicated that the predicted-to-actual similarity was at chance (akin to chance decoding using classifiers). We tested for multiple comparisons using permutation testing[15] for every region-to-region mapping, and significance was assessed using FWE-corrected p-values with $p<0.05$. Note that to avoid circularity for region-to-region information transfer mapping, any vertices in a source region that fell within a 10mm radius of the to-be-predicted target region (e.g., an adjacent region) would not contribute any activity flow to the to-be-predicted target region (see FC estimation Methods section for details).

Given the visual sparsity of the region-to-region information transfer mapping visualization, we opted to down sample our matrix to provide a simpler visualization to assess how pairs of regions transfer information between and within functional networks

(Fig. 6C,E,G). Thus, we computed the percent of statistically significant transfers for every pair of networks. This allowed us to better visually assess how region-to-region information transfer mappings may have been influenced by underlying network organization. To compute the percent of statistically significant transfers, we counted the number of significant transfers between every pair of networks and divided that by the total number of possible transfers within that network-to-network configuration. To characterize the generality with which information transfer mappings occurred between specific network configurations, we computed the number of rule domains in which each network configuration contained at least one region-to-region transfer (Fig. 6H). In other words, we took the matrices in Fig. 6C,E,G and binarized them with a 1 if a cell had a greater than 0 percentage of transfers, and a 0 otherwise. We then summed these matrices element-wise to obtain the number of rule domains each network configuration had a successful information transfer in. To assess the number of rule domains each network contained at least one successful source region, we took the percent of significant transfers from each network to any other region in the brain (a 7-element array) and then binarized the array for each rule domain. We then summed across the three arrays (one for each rule domain) to obtain the number of rule domains each network had at least one successful source region used for information transfer (Fig. 6I).

Lastly, to visualize the anatomical locations of the source regions for information transfer, we computed the percent of significant transfers from each cortical region for each rule domain (Fig. 7). Percentages were obtained by taking the number of successful transfers from a region, and dividing it by total number of possible transfers (i.e., 359 other regions). We then plotted each of these percentages on the cortical surface using Connectome Workbench software (version 1.2.3) for each rule domain[18].

Network-to-network information transfer mapping

Network-to-network information transfer mapping in both the computational model (Fig. 4E) and empirical data (Supplementary Fig. 1C,D,E) was performed in the same computational framework as above, though instead of predicting region-level activation patterns using vertex-level activation patterns, network-level activation patterns were predicted using region-level activations (averaging across vertices within a given region). In other words, when predicting a target network B's region-level activation pattern, we computed the dot product between a source network A's region-level activity vector and the region-to-region resting-state FC matrix between regions in network A and B. We then submitted our 128 task block predictions for network B to our information transfer mapping procedure, as described above. This was repeated for every pair of the seven functional networks defined by our community-detection algorithm, resulting in 7-by-7 network-to-network mappings which were visualized as a 7x7 matrix (Supplementary Fig. 1C,D,E). We tested for multiple comparisons using FWE-correction for every network-to-network mapping within a rule domain, and significance was assessed using the FWE-corrected p-values of $p<0.05$.

Permutation testing of FC topology

We hypothesized that the precise topology of resting-state FC described the baseline architecture of information processing during task states. Thus, to ensure that our information transfer mapping procedure depended on resting-state FC topology, we

performed permutation testing, shuffling the network-to-network FC topology prior to performing information transfer mapping. Due to computational cost, we limited this control analysis to network-to-network information transfer mapping.

For each subject, we permuted the network-to-network resting-state FC prior to applying the information transfer mapping procedure for every pair of networks. More specifically, each network's connectivity was permuted within-network, such that no FC values from one network was ever moved to another network. This helped ensure that the permutations only altered the network-to-network FC topology, such that (for example) the overall mean level of FC between the networks was never altered across the permutations. To correct for multiple comparisons, a single permutation cycle involved permuting the FC topology for every pair of networks, for all subjects. We then performed a group t-test for every pair of network-to-network information transfers, extracting the maximal t-statistic across all network-to-network comparisons. We ran 1000 of these permutation cycles, obtaining the maximal t-statistic for each permutation. This formed a null distribution of the maxima across the family of tests (i.e., all possible network-to-network information transfers), thus controlling for FWE[15]. Using our permutation distribution, we computed FWE-corrected p-values with a one-tailed test, i.e., $p = P(X > ite)$, where ite corresponds to the true information transfer estimate, and X as the null distribution of maximal t-statistics. Statistical significance was then assessed using a FWE-corrected threshold of p<0.05.

Behavioral relevance of information transfers

To characterize the behavioral relevance of information transfers, we performed a within-subject analysis to decode task performance using miniblock-by-miniblock information transfer estimates. We first sought to ensure that baseline miniblock information estimates could decode miniblock task performance within subjects prior to the information transfer mapping procedure. We defined miniblock information estimates as

$$\boldsymbol{IE}_{X_k} = \boldsymbol{match}_{X_k} - \boldsymbol{mismatch}_{X_k} \qquad (11)$$

where $\boldsymbol{IE}_{X_k}$ corresponds to the information estimate of rule domain X during miniblock k, $\boldsymbol{match}_{X_k}$ corresponds to the matched task-rule condition similarity of rule domain X during miniblock k, and $\boldsymbol{mismatch}_{X_k}$ corresponds to the averaged rank correlation of miniblock k's activation pattern to the mismatched task-rule conditions.

To perform a given task, knowledge of all three rule domains (i.e., logic, sensory, and motor rule domains) is required. Thus, we constructed a decoding model with logistic regression, training the model to decode the task performance of a given miniblock using the information estimates of a given brain region across all three rule domains. The model was tested using cross-validation in MATLAB using the glmfit function (with the logit link function), and was formulated as

$$\vec{y}_{accuracy} = f(\beta_0 + \beta_1 \boldsymbol{X}_L + \beta_2 \boldsymbol{X}_S + \beta_3 \boldsymbol{X}_M) \qquad (12)$$

where $\vec{y}_{accuracy}$ corresponds to the vector containing task accuracy for all miniblocks, $\boldsymbol{x}_L$, $\boldsymbol{x}_S$, $\boldsymbol{x}_M$ correspond to the regressors for logic, sensory, and motor information estimates, respectively, β_0 corresponds to the training bias (which accounts for the imbalance of the correct:error trial ratio), and $\beta_1, \beta_2, \beta_3$ correspond to the estimated model coefficients for the logic, sensory, and motor information estimates, respectively. The link function f corresponds to the sigmoid function, defined as

$$f(x) = \frac{1}{1 + e^{-x}} \qquad (13)$$

Miniblocks with over 50% of trials performed correctly were characterized as a 1, and 0 otherwise.

To test our model, we used cross-validation to predict the binarized accuracy of held-out data. However, to account for the imbalanced training data (on average, subjects performed 85% of trials correctly), we removed the intercept term β_0 to center our predictions (as computed by a sigmoid function) at 0.5. Thus, our predictions on held-out data were computed as probabilities by the equation

$$g(x_L, x_S, x_M) = f(\beta_1 x_L + \beta_2 x_S + \beta_3 x_M) \qquad (14)$$

where $g \in (0, 1)$, and accuracies were predicted/classified by the equation

$$\boldsymbol{G}_{decoder}\big(g(\boldsymbol{x})\big) = \begin{cases} 1 & g(\boldsymbol{x}) > 0.5 \\ 0 & g(\boldsymbol{x}) < 0.5 \end{cases} \qquad (15)$$

, where $\boldsymbol{G}_{decoder}$ generates predictions for miniblocks with greater than 50% task performance as 1, and 0 otherwise.

Given that region-to-region information transfers consistently occurred between regions in the FPN and CON across all three rule domains (Fig. 6H), we constrained our search to those networks. We applied our decoding model to all regions within the FPN and CON across subjects. For each region, we applied one-sided t-tests against chance (50%), and corrected for multiple comparisons using FWE-correction permutation tests[15]. We identified a single FPN region in the LPFC (LH region 80 in the Glasser et al. atlas; Supplementary Figure 5) whose baseline information estimates predicted miniblock task performance.

We subsequently tested whether information transfer estimates from the LPFC region could predict task performance. We applied the decoding model to information transfer estimates across all rule domains (instead of baseline information estimates) for all information transfers from the LPFC region to all other FPN and CON regions. (We used the LPFC region here as the "source" region, obtaining decoding accuracies from that region to all other FPN/CON regions.) We performed one-sided t-tests against chance (50%) for each information transfer, and corrected for multiple comparisons using FWE-correction permutation tests[15]. We identified a single information transfer from the LPFC to the OFC (LH region 91; both FPN regions) that survived multiple corrections with an FWE-corrected p<0.05. Surface visualizations for Supplementary Figure 5 were made using Connectome Workbench software (version 1.2.3)[18].

Supplementary References

1. Cole, M. W., Bagic, A., Kass, R. & Schneider, W. Prefrontal Dynamics Underlying Rapid Instructed Task Learning Reverse with Practice. *J. Neurosci.* **30,** 14245–14254 (2010).
2. Huth, A. G., Heer, W. A. De, Griffiths, T. L., Theunissen, F. E. & Jack, L. Natural speech reveals the semantic maps that tile human cerebral cortex. *Nature* **532,** 453–458 (2016).
3. Nishimoto, S. *et al.* Reconstructing visual experiences from brain activity evoked by natural movies. *Curr. Biol.* **21,** 1641–1646 (2011).
4. Simony, E. *et al.* Dynamical reconfiguration of the default mode network during narrative comprehension. *Nat. Commun.* **7,** 1–13 (2016).
5. Schneider, W., Eschman, A. & Zuccolotto, A. *E-Prime: User's guide*. (Psychology Software Incorporated, 2002).
6. Power, J. D., Barnes, K. A., Snyder, A. Z., Schlaggar, B. L. & Petersen, S. E. Spurious but systematic correlations in functional connectivity MRI networks arise from subject motion. *Neuroimage* **59,** 2142–2154 (2012).
7. Glasser, M. F. *et al.* The minimal preprocessing pipelines for the Human Connectome Project. *Neuroimage* **80,** 105–124 (2013).
8. Cole, M. W., Ito, T., Bassett, D. S. & Schultz, D. H. Activity flow over resting-state networks shapes cognitive task activations. *Nat. Neurosci.* (2016). doi:10.1038/nn.4406
9. Blondel, V. D., Guillaume, J.-L., Lambiotte, R. & Lefebvre, E. Fast unfolding of communities in large networks. *J. Stat. Mech. Theory Exp.* **10008,** 6 (2008).
10. Jutla, I. S., Jeub, L. G. S. & Mucha, P. J. A generalized Louvain method for community detection implemented in MATLAB. *URL http//netwiki. amath. unc. edu/GenLouvain* (2011).
11. Traud, A. L., Kelsic, E. D., Mucha, P. J. & Porter, M. a. Comparing Community Structure to Characteristics in Online Collegiate Social Networks. *SIAM Rev.* **53,** 17 (2011).
12. Power, J. D. *et al.* Functional Network Organization of the Human Brain. *Neuron* **72,** 665–678 (2011).
13. Gordon, E. M. *et al.* Generation and Evaluation of a Cortical Area Parcellation from Resting-State Correlations. *Cereb. Cortex* (2014). doi:10.1093/cercor/bhu239
14. Yeo, B. T. T. *et al.* The organization of the human cerebral cortex estimated by intrinsic functional connectivity. *J. Neurophysiol.* **106,** 1125–1165 (2011).
15. Nichols, T. E. & Holmes, A. P. Nonparametric permutation tests for functional neuroimaging: A primer with examples. *Hum. Brain Mapp.* **15,** 1–25 (2002).
16. Barral, J. & Reyes, A. D. Synaptic scaling rule preserves excitatory–inhibitory balance and salient neuronal network dynamics. *Nat. Neurosci.* (2016). doi:10.1038/nn.4415
17. Oosterhof, N. N., Wiestler, T., Downing, P. E. & Diedrichsen, J. A comparison of volume-based and surface-based multi-voxel pattern analysis. *Neuroimage* **56,** 593–600 (2011).
18. Glasser, M. F. *et al.* The Human Connectome Project's neuroimaging approach. *Nat. Neurosci.* **19,** 1175–87 (2016).
